# Suppression of ATAD2 inhibits hepatocellular carcinoma progression through activation of p53- and p38-mediated apoptotic signaling

**DOI:** 10.18632/oncotarget.6152

**Published:** 2015-10-19

**Authors:** Wen-Jing Lu, Mei-Sze Chua, Samuel K. So

**Affiliations:** ^1^ Asian Liver Center, Department of Surgery, Stanford University School of Medicine, Stanford, CA, USA

**Keywords:** ATAD2, apoptosis, mutant p53, liver cancer, targeted therapy

## Abstract

The ATPase family, AAA domain containing 2 (ATAD2) is highly expressed in multiple cancers. We aim to understand the clinical and biological significance of ATAD2 over-expression in hepatocellular carcinoma (HCC), as a means to validate it as a therapeutic target in HCC. We demonstrated that ATAD2 was over-expressed in HCC patients, where high ATAD2 levels were significantly correlated with aggressive phenotypes such as high AFP levels, advanced tumor stages, and vascular invasion. Using RNA interference, suppression of ATAD2 in HCC cell lines decreased cell viability, migration, and invasion, and induced apoptosis *in vitro*. Furthermore, we identified p53 and p38 as key proteins that mediate apoptosis induced by ATAD2 suppression. In HCC cells, we demonstrated that ATAD2 directly interacted with MKK3/6, which prevented p38 activation and therefore inhibited p38-mediated apoptosis. *In vivo,* suppression of ATAD2 impaired the growth of HepG2 and Hep3B subcutaneous xenografts, accompanied by enhanced apoptosis and p-p53 and p-p38 levels. Our results validate that ATAD2 is an important negative regulator of apoptosis, and that neutralizing its activity has promising anti-tumor effects in HCC cells.

## INTRODUCTION

Hepatocellualr carcinoma (HCC), the primary malignancy of the adult liver, is a global health problem with unmet medical needs. Current global cancer statistics rank it as the second leading cause of cancer related death, with a high mortality-to-incidence ratio of 0.95 (2012 Globocan (WHO IARC). HCC patients are often detected when the disease is in its advanced stages, when there are limited therapeutic options. Together with inherent drug resistance and high recurrence rates, HCC patients often have poor prognosis with short overall survival time following diagnosis. Despite recent advances in the molecular pathogenesis of HCC [1], there is still a lack of molecularly targeted therapeutic approaches for the effective treatment of HCC, with sorafenib being the only drug for treating advanced HCC that has been approved by the USA Food and Drug Administration in the past decade [2]. There is therefore still an imperative need to identify clinically and biologically relevant molecular targets of HCC that can be exploited for therapeutic purposes.

ATPase family AAA domain-containing protein 2 (ATAD2) is a remarkably conserved protein that is located primarily in the cell nucleus. Its protein structure consists of two AAA domains and one bromodomain, indicating that the functions of ATAD2 are related to genome regulation, such as by acting on chromatin structure and function [3]. ATAD2 was found to be an abundantly expressed epigenetic factor in male germ cells, as well as in a wide variety of solid tumors [4]. Specifically, ATAD2 up-regulation was observed in 760 cancer samples of 14 different origins, compared to 112 normal somatic tissues [4], and this up-regulation was correlated with poor prognosis in lung cancer [4, 5], breast cancer [6], osteosarcoma [7], and HCC [8, 9]. Functional studies revealed that suppression of ATAD2 induced spontaneous cell apoptosis [4, 10]. When ATAD2 is activated, it initiates a loop of transcriptional stimulation of target genes, including itself, to enhance cell proliferation and resistance to apoptosis [11]. Additionally, ATAD2 functions as a co-activator of the estrogen receptor [12] and the androgen receptor [10], implicating it as an oncogenic protein in hormone-related cancers such as breast cancer and prostate cancer. ATAD2 also functions as a cofactor with other transcription factors like E2F1-4 [13] and c-Myc [14, 15] to regulate specific set of genes which have oncogenic functions.

Our study was aimed at studying the clinical significance of ATAD2 over-expression in HCC, and to validate it as a potential therapeutic target using *in vitro* and *in vivo* HCC models. Additionally, we further studied the effects of ATAD2 on cell apoptosis and the signaling pathways involved, in order to obtain further insights into the underlying mechanisms, as well as to identify possible ways of interfering with this function.

## RESULTS

### Overexpression of ATAD2 is correlated with aggressive HCC phenotypes

**Figure 1:**
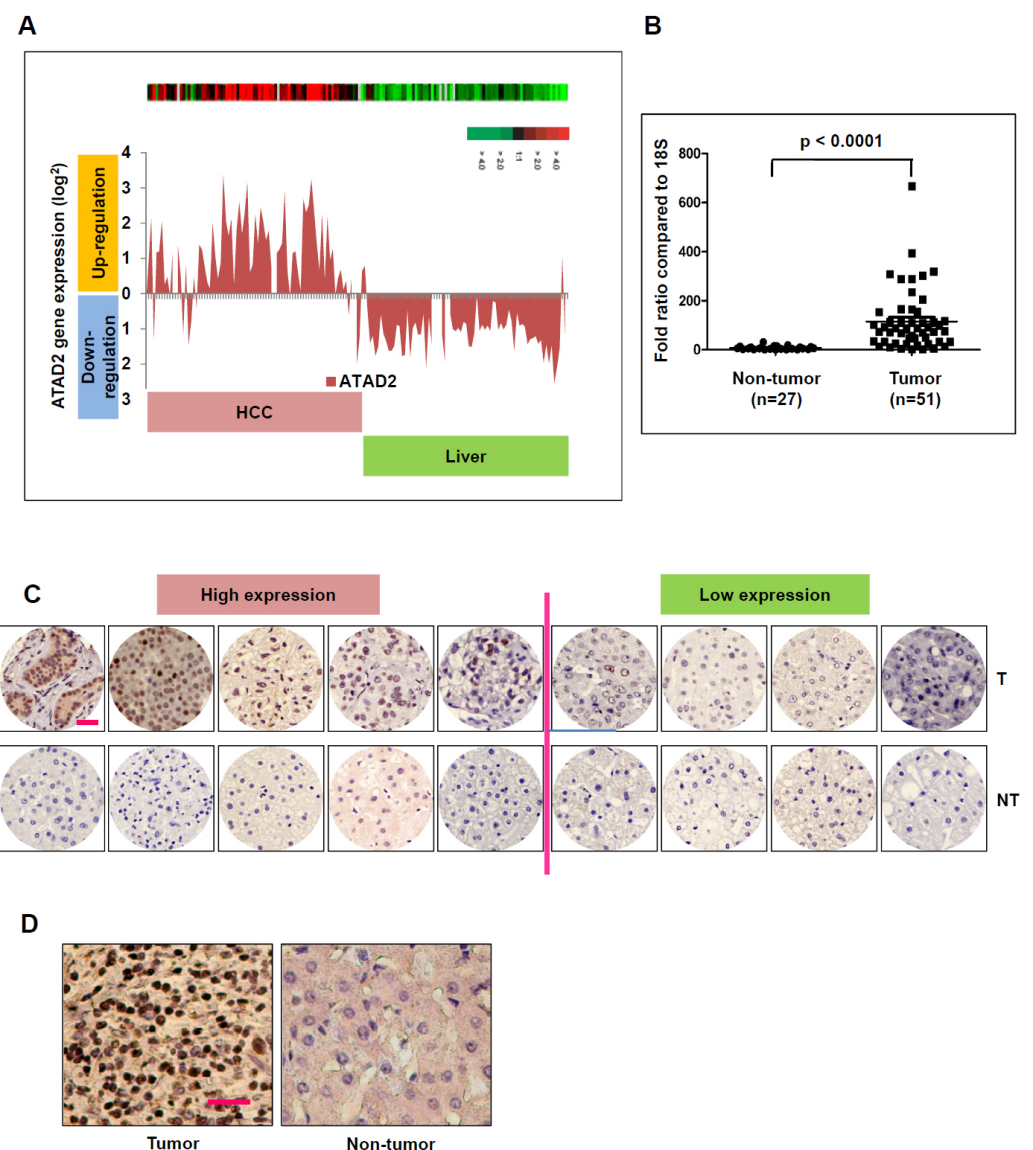
ATAD2 is over-expressed in human HCC samples **A.** ATAD2 transcript is up-regulated in HCC tumors compared to matched non-tumor livers in 75 HCC patients in Cohort 1. **B.** Real-time qPCR measurement of ATAD2 transcript confirmed its significant over-expression in HCC tumors compared to matched non-tumor livers in Cohort 1. (***p < 0.0001) **C.** Immunohistochemical staining of ATAD2 protein in 9 pairs of HCC tumor (T) and matched non-tumor (NT) livers from patients in Cohort 1. (200X magnification; scale bar: 50 μm). Low expression: positive cells present in < 50% of the entire area; High expression: positive cells present in > 50% of the entire area. **D.** Representative immunohistochemical images of ATAD2 protein expression in 82 cases of HCC patients in Cohort 2, represented on tissue array (200X magnification; scale bar: 50 μm).

We extracted ATAD2 transcript expression data from our earlier gene expression profiling study of HCC patients [16], and observed significant over-expression of ATAD2 transcript in HCC tissues compared to matched adjacent non-tumor liver in 75 HCC patients (Figure 1A; *p* < 0.05). Correlation analysis of ATAD2 transcript expression level (high - above average; low - below average) with clinical pathological data of these 75 HCC patients suggested that ATAD2 expression was significantly associated with high AFP level (*p* < 0.0353), advanced tumor stages (*p* < 0.0358), and vascular invasion (*p* < 0.0211) (Table 1). Thus, high ATAD2 expression was correlated with more aggressive HCC phenotypes. Using tissue samples (51 HCC and 27 non-tumor liver) from the same patient cohort, we validated over-expression of ATAD2 transcript in HCC using TaqMan real-time semi-quantitative PCR (*p* < 0.0001) (Figure 1B). Immunohistochemical (IHC) staining of a small subset of this patient cohort (*n* = 9; HCC and paired non-tumor liver) further validated the over-expression of ATAD2 protein in five out of these nine HCC patients (55.6%) (Figure 1C). IHC staining of an independent sample set (*n* = 82) represented on tissue microarrays confirmed the over-expression of ATAD2 protein in 58.5% (48/82) of HCC patients (representative images shown in Figure 1D; IHC scores are shown in Supplementary Table 1).

**Table 1:** Clinical correlation between ATAD2 mRNA expression level and clinico-pathological parameters of HCC patients (*n* = 75)

	ATAD2 expression^a^		
Variable	High	Low	*p* value
			
Sex			0.5829
Male	31	28	
Female	7	9	
			
Age			0.4686
<60	27	23	
>=60	11	14	
			
HBsAg			0.1373
Negative	4	9	
Positive	34	28	
AFP, ng/ml			0.0353*
<200	12	18	
>=200	18	8	
			
Tumor size, cm			0.632
<5	15	12	
>=5	23	25	
Histological differentiation			0.0897
Well	3	8	
Moderate/Poor	27	18	
			
Tumor Stage			0.0358*
Early (I, II)	11	20	
Late (III, IV)	27	17	
Vascular invasion			0.0211*
Absent	14	24	
Present	24	13	
			
Microsatellite			0.1077
Absent	15	22	
Present	23	15	
Recurrence			1
Absent	21	20	
Present	17	17	

a The cutoff is the mean value of ATAD2 mRNA expression.

* significance, *p* < 0.05)

### Suppression of ATAD2 inhibited HCC progression *in vitro*

**Figure 2:**
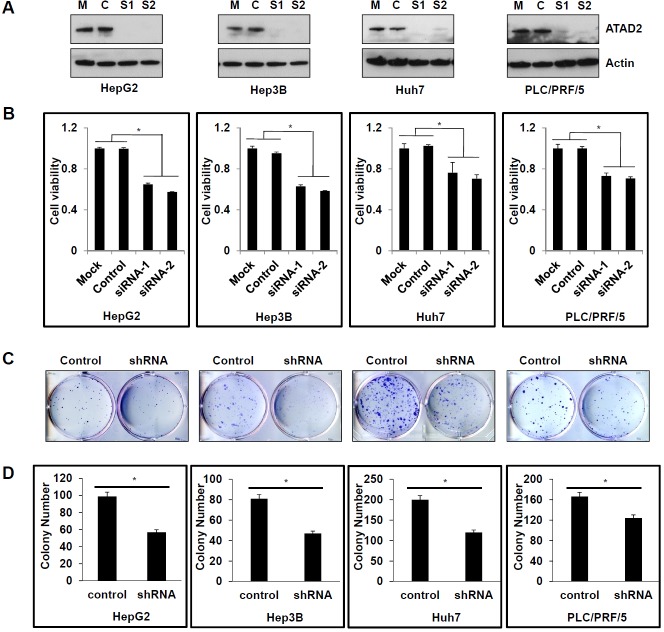
Suppression of ATAD2 inhibited HCC cell viability and colony formation **A.** Western blot detection of ATAD2 protein in HCC cells that are mock (M) transfected with transfection reagent only; Control (C)-transfected with control siRNA, or transfected with ATAD2-siRNA1 (S1) or ATAD2-siRNA2 (S2). Actin was used as the internal loading control. **B.** ATAD2 suppression in HCC cell lines decreased cell viability compared to mock or control siRNA cells (*p < 0.05). **C.** HCC cells stably expressing ATAD2 shRNA showed decreased colony formation ability compared to control cells. **D.** Quantification of colony formation in HCC cells stably expressing ATAD2 shRNA. Triplicate independent experiments were performed; *p < 0.05.

To further examine the functional significance of ATAD2 in HCC, two ATAD2-specific siRNAs were used to silence the expression of ATAD2 in four HCC cell lines that have high endogenous levels of ATAD2 (HepG2, Hep3B, Huh7, and PLC/PRF/5). Knock down efficiency was confirmed by Western blot, showing at least 90% suppression of ATAD2 compared to mock or control cells (Figure 2A). Additionally, transient suppression using ATAD2-specific siRNAs significantly decreased cell viability (Figure 2B) and cell growth rate (Supplementary Figure 1), whereas stable suppression using ATAD2 shRNA significantly decreased colony formation (Figure 2C and 2D) in all four cell lines.

**Figure 3:**
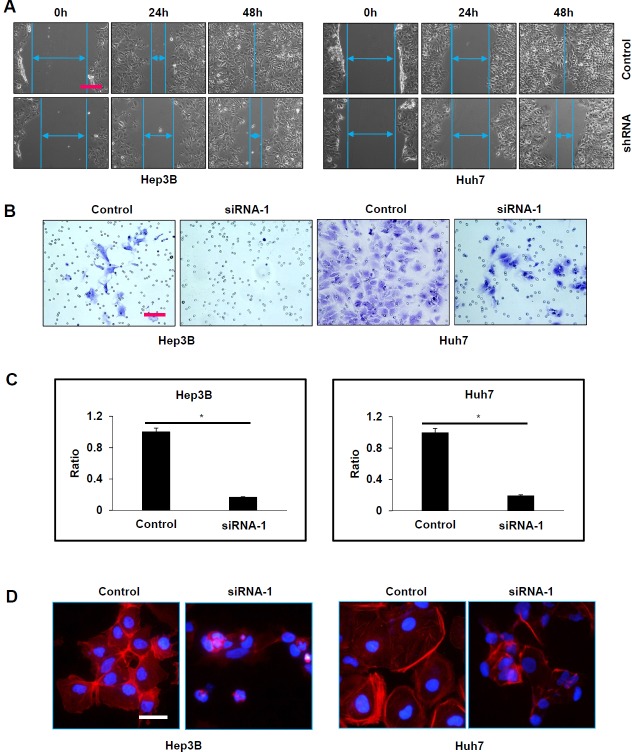
Suppression of ATAD2 impaired HCC cell mobility and invasion **A.** In wound healing assay, both Hep3B and Huh7 cells that stably express ATAD2 shRNA migrated slower and were able to close the wound slower than control cells (100X magnification; scale bar: 250 μm). **B.** In matrigel invasion assay, the number of invaded cells was significantly increased in HCC cells transfected with ATAD2 siRNA-1 compared to control cells (200X magnification; scale bars: 50 μm). **C.** Quantification of cells that invaded through the matrigel-coated membrane (200X magnification; scale bars: 10 μm). (*p < 0.05) **D.** HCC cells transfected with ATAD2 siRNA-1 demonstrate disrupted stress fiber network. Stress fiber (polymerized actin) was identified by phalloidin staining (red) in Hep3B and Huh7 cells (200X magnification; scale bar: 50 μm).

Since ATAD2 over-expression was observed to be significantly correlated with vascular invasion in HCC patients, we studied the effects of ATAD2 suppression on cell motility and invasion using the wound-healing assay and the matrigel invasion assay, respectively. In the wound-healing assay, Hep3B and Huh7 cells with stable suppression of ATAD2 showed slower migration towards the wound compared with control cells (Figure 3A). Moreover, Hep3B and Huh7 cells with transient suppression of ATAD2 showed decreased invasiveness compared with control cells (Figure 3B and 3C). Additionally, we found that HCC cells with ATAD2 suppression also showed reduced stress fiber formation, as detected by phalloidin staining, suggesting that ATAD2 may participate in cytoskeletal reorganization of cancer cells (Figure 3D). Our *in vitro* results suggest that cells with suppressed ATAD2 levels were less tumorigenic and metastatic, indicating a role of ATAD2 in HCC progression.

### Apoptosis induced by ATAD2 suppression is dependent on p53 and/or p38

**Figure 4:**
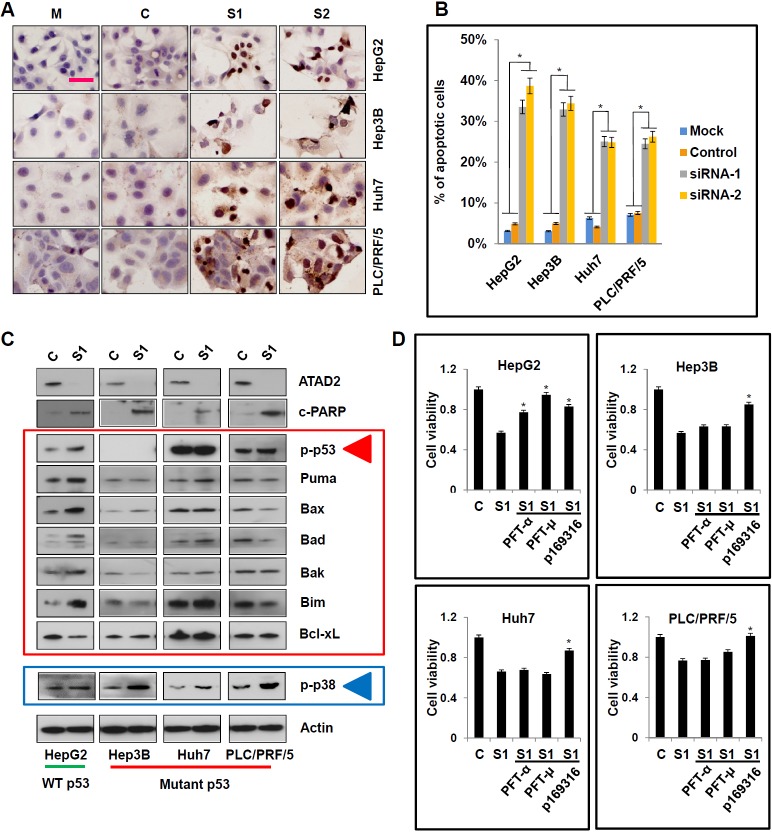
Suppression of ATAD2 facilitated p53- and p38-dependent apoptotic signaling in HCC cells **A.** *In situ* TUNEL staining for the detection of apoptotic cells after suppression of ATAD2 using siRNA (200X magnification; scale bars: 50 μm). **B.** Quantification of apoptotic cells in HCC cells treated with ATAD2 siRNA-1 (*p < 0.05). **C.** Suppression of ATAD2 in HCC cells induced p53- and p38-mediated apoptotic pathways, evidenced by increased p-p53, p-p38, Puma, Bax, Bad, Bak, Bim, and decreased in Bcl-xL. **D.** When HCC cells were co-treated with ATAD2 siRNA-1 and with p53 inhibitor (PFT-α and PFT-μ) or p38 inhibitor (p169316), apoptosis was partially reversed when compared to control group.

To determine the mechanism(s) underlying the decrease in cell viability caused by ATAD2 suppression, we used TUNEL staining to detect apoptosis in HCC cells treated with ATAD2 siRNA. We observed 25-35% of positive nuclear TUNEL staining in HCC cells treated with ATAD2 siRNA, but not in the control or mock group cells (Figure 4A and 4B). Using Western blotting, we observed that ATAD2 siRNA activated the p53-Bcl-_2_ family proteins in HepG2 cells with wild-type p53, but not in other HCC cells (Hep3B, Huh7, PLC/PRF/5) with mutant p53 (Figure 4C). Specifically, in HepG2 cells treated with ATAD2 siRNA, phosphorylated p53 and the pro-apoptotic proteins Puma, Bax, Bad, Bak, and Bim were increased, whereas the anti-apoptotic protein Bcl-xL expression was decreased. Our results suggested that other alternative apoptotic pathways may be activated in HCC cells with mutant p53. Indeed, we observed that ATAD2 suppression activated p38 (evidenced by increased p-p38) in HCC cells with mutant p53 (Figure 4C).

When HCC cells were co-treated with ATAD2 siRNA and specific inhibitors of p53 (PFT-α and PFT-μ) or p38 (p168316), we observed that both p53 and p38 inhibitors could reverse the decrease in cell viability caused by ATAD2 suppression in HepG2 cells (Figure 4D). However, only the p38 inhibitor could reverse this effect in the other three HCC cell lines with mutant p53 (Figure 4D). These results indicated that apoptosis induced by ATAD2 suppression is mediated by both p53-Bcl-2 and p38 pathways, depending on specific p53 status of the cell lines.

### ATAD2 directly interacts with MKK3 and MKK6, two dual-specificity protein kinases that activate p38 phosphorylation

**Figure 5:**
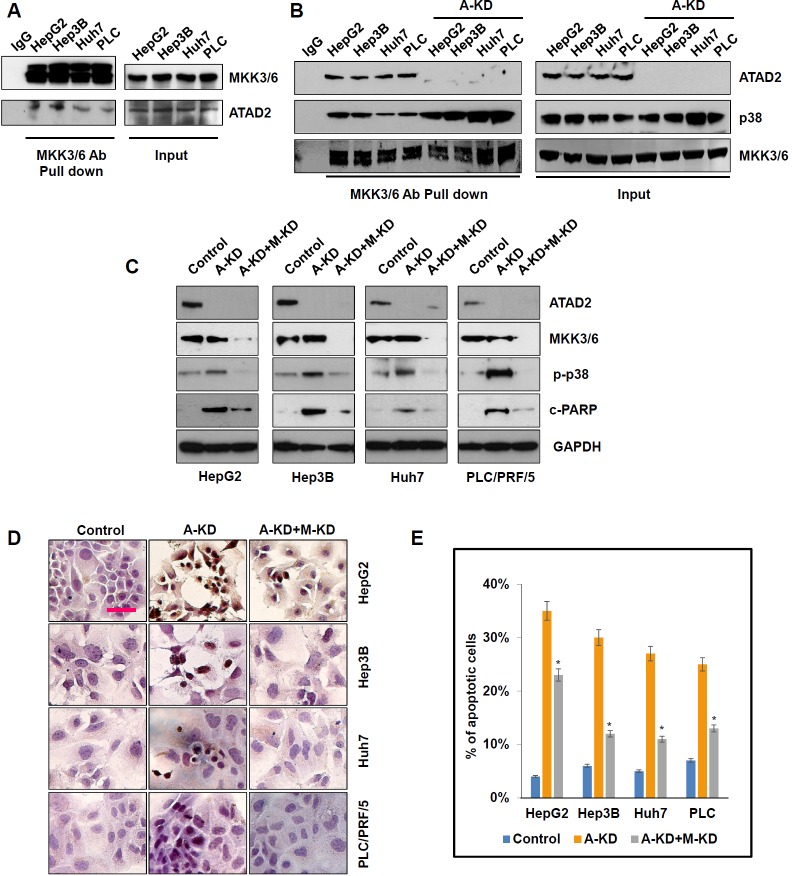
ATAD2 directly interacted with MKK3/6 to abrogate MKK3/6-induced phosphorylation of p38 **A.** Co-immunoprecipitation assay showing interaction of ATAD2 with MKK3/6 in HCC cells. **B.** Suppression of ATAD2 using siRNA-1 (A-KD) enhanced MKK3/6 binding to p38 as determined by co-immunoprecipitation. **C.** Western blot indicating that co-treatment of HCC cells with ATAD2 siRNA-1 (A-KD) and MKK3/6 siRNA (M-KD) decreased p-p38 and c-PARP levels. **D.** TUNEL staining showing that the number of positively stained apoptotic cells decreased in HCC cells that were co-treated with ATAD2 siRNA-1 (A-KD) and MKK3/6 siRNA (M-KD) compared to control cells (200X magnification; scale bar: 50 μm). **E.** Quantification of apoptotic cells detected in **D.** (*p < 0.05).

MKK3 and MKK6 are two major factors that regulate p38 phosphorylation. To understand how ATAD2 regulates p38 phosphorylation in HCC cells, we first determined whether ATAD2 interacts with MKK3/6 by co-immunoprecipitation. In all four HCC cell lines tested, MKK3/6 was pulled-down by ATAD2, indicating direct interaction of these proteins (Figure 5A). When ATAD2 was suppressed in these cell lines, we observed enhanced MKK3/6 and p38 complex formation (Figure 5B), suggesting that ATAD2 abrogates interaction between p38 and MKK3/6. When HCC cells were co-transfected with ATAD2 siRNA and MKK3/6 siRNA, the additional suppression of MKK3/6 reduced the enhancement of p-p38, and also rescued cells from apoptosis (measured by c-PARP) caused by ATAD2 suppression in all four HCC cell lines, especially those with mutant p53 (Figure 5C). TUNEL staining results were consistent with Western blot results of c-PARP levels (Figure 5D and 5E).

### Suppression of ATAD2 *in vivo* inhibited tumor growth through inducing apoptosis of HCC cells

**Figure 6:**
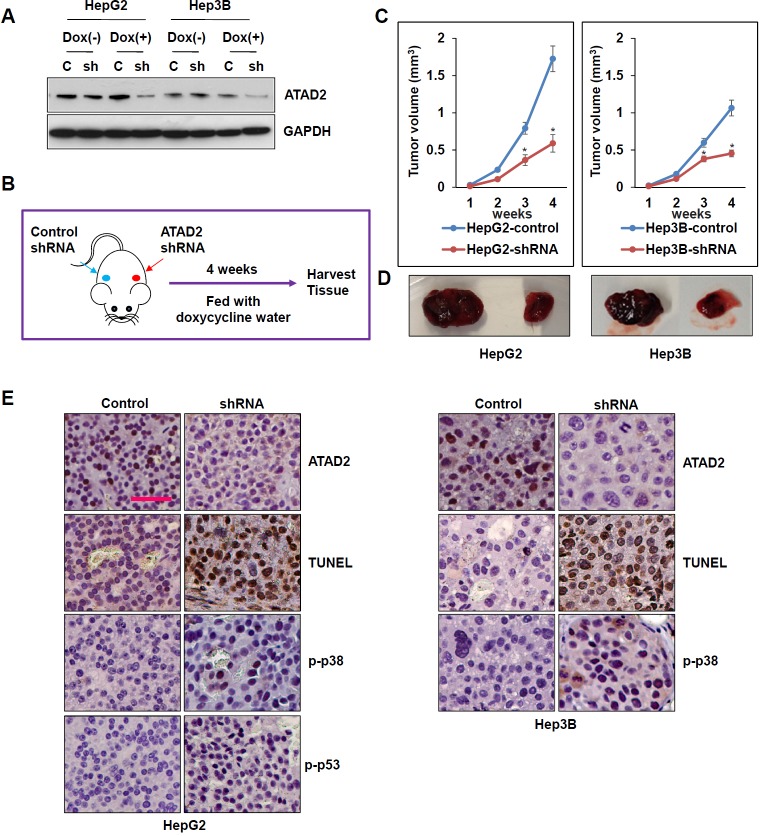
Suppression of ATAD2 impaired growth of HepG2 and Hep3B subcutaneous xenografts **A.** ATAD2 expression in HepG2 and Hep3B cells stably expressing ATAD2 shRNA was detected by Western blotting. GAPDH was used as an internal loading control. **B.** Schematic model of *in vivo* experiment. **C.** Growth curves showing the effect of ATAD2 suppression in the growth of HepG2 and Hep3B subcutaneous xenografts (*n* = 5 in each group). (*p < 0.05). **D.** Representative images showing harvested tumors derived from HepG2 and Hep3B cells stably expressing control shRNA or ATAD2 shRNA, 4 weeks post-inoculation. **E.** Immunohistochemical staining of paraffin sections of xenograft tissues showing that ATAD2 suppression was accompanied by increased TUNEL staining, and increased p-p53 and p-p38 levels (200X magnification; scale bar: 50 μm).

To further examine the effects of ATAD2 suppression *in vivo*, we first generated lentivirus-derived doxycycline *tet-*on control and ATAD2-shRNA expression vectors. HepG2-*Luc*(+) and Hep3B*-Luc*(+) cells were then transduced with these vectors and selected for stable ATAD2-shRNA expression, and used to establish xenografts in nude mice. *In vitro,* cell lines that stably express ATAD2-shRNA showed greatly reduced expression of ATAD2 after doxycycline treatment (Figure 6A). Cells stably expressing ATAD2-shRNA or control shRNA were then inoculated subcutaneously into the shoulder of nude mice, which were fed water containing doxycycline (Figure 6B). After four weeks, the tumor volumes of ATAD2-shRNA groups (in both HepG2 and Hep3B xenografts) were significantly smaller than that of control groups (Figures 6C-6D; *p* < 0.05). At the end of the experiment, mice were sacrificed and tumor tissues were harvested for detection of apoptosis. TUNEL staining showed markedly increased apoptotic cells in xenografts derived from ATAD2-shRNA cells (Figure 6E). Moreover, ATAD2 suppression and apoptosis were associated with increased p-p53 (in HepG2 cells) and p-p38 expressions (in HepG2 and Hep3B cells) (Figure 6E). Our *in vivo* results indicated that suppression of ATAD2 inhibited HCC tumor growth through inducing p53- and p38-mediated apoptosis.

## DISCUSSION

The over-expression of ATAD2 has been reported in multiple solid tumors in humans, suggesting its universal role in carcinogenesis independent of tissue types. Our study shows that in HCC, ATAD2 is a feasible therapeutic target, due to its clinical association with aggressive HCC phenotypes, and its biological functions in promoting tumor cell growth and invasion. Specifically, we showed that over-expression of ATAD2 correlated significantly with high AFP levels, advance tumor stages, and vascular invasion. The suppression of ATAD2 expression elicited anti-tumor functions, including inhibition of HCC cell proliferation, migration, and invasion, while inducing apoptosis *in vitro*. Consistently, ATAD2 suppression in subcutaneous HCC xenografts delayed tumor cell growth, accompanied by apoptosis induction.

Our results are consistent with recently reported clinical associations of ATAD2 expression and its functions in HCC. Hwang *et al* [8] and Wu *et al* [9] observed that ATAD2 expression correlated with poor survival, consistent with an oncogenic function. Consistent with our data, ATAD2 depletion by RNA interference was reported to reduce HCC cell invasion and proliferation *in vitro* [9], which was associated with marked up-regulation of APC and down-regulation of CTNNA1. Functional studies further suggested an interaction between ATAD2 and the Hedgehog (Hh) pathway, whereby ATAD2 cooperates with the c-Myc gene to regulate the expression of SMO and Gli, activating the Hh pathway and inducing an active feedback of the Hh pathway [14].

**Figure 7:**
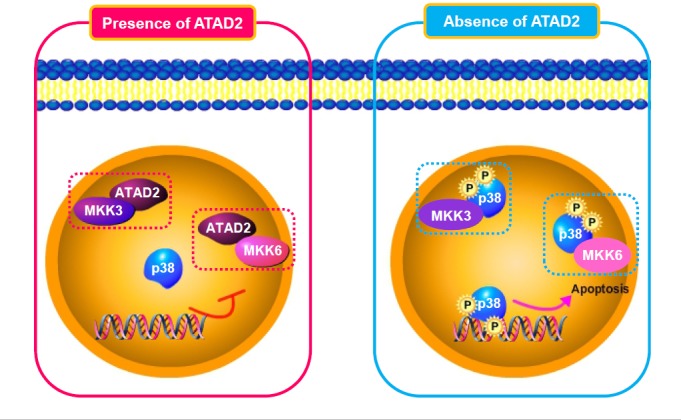
Schematic model of ATAD2 interaction with MKK3/6 and subsequent regulation of p38 phosphorylation-mediated apoptosis

Our study provides further mechanistic insights by revealing that apoptosis induced by ATAD2 suppression was mediated through p53 and/or p38, depending on the cell context. In HCC cells with wild-type p53, ATAD2 suppression activated both p53- and p38-mediated apoptosis; in HCC cells with mutant p53, ATAD2 suppression only activated p38-mediated apoptosis. Furthermore, we demonstrated that ATAD2 directly interacted with p38 phosphorylases MKK3/6 to abrogate their interactions with p38; the inability to activate p38 then caused cells to escape apoptosis. Conversely, the suppression of ATAD2 increased interactions of MKK3/6 with p38, leading to p38 activation and subsequently apoptosis (Figure 7). Thus, the inhibitions of ATAD2 expression or of its interactions with MKK3/6 are potential therapeutic strategies by which apoptosis can be stimulated to achieve anti-tumor effects. Additionally, our finding that ATAD2 can induce apoptosis through p38 in cancer cells with mutant p53 offers a new approach for the clinical management of p53 mutant tumors.

Apoptosis mediated by p53 serves as a vital tumor suppression mechanism to eliminate unstable cells. However, in many cancers including HCC, this apoptotic pathway is often deregulated due to p53 mutations [17, 18] or binding to inhibitory proteins of p53 (such as Parc and mortalin) [19, 20]. The accumulation of cytoplasmic mutant p53 also blocks the functions of wild-type p53 in a dominant-negative manner [21]. In HCC, defects in the p53 pathway are frequently associated with cell proliferation, metastasis, vascular invasion, tumor recurrence, and poor prognosis. Thus, the ability to reactivate mutant p53 or to activate alternative apoptotic pathways offers a feasible therapeutic approach in such cancers. We demonstrated that the activation of p38-mediated apoptosis through ATAD2 suppression is an example of such an alternative apoptotic pathway in HCC cells.

The major function of p38 mitogen-activated protein kinase (MAPK) is to control cell differentiation, prevent proliferation, and induce apoptosis [22]. The p38 phosphorylases MKK3 and MKK6 are two major factors that regulate p38 activation through phosphorylation in response to cellular stress and cytokines [23]. In double knockout cells for both MKK3 and MKK6 (MKK3−/− MKK6−/−), p38 cannot be phosphorylated and its subsequent actions are lost [24]. Several studies implicate p38 as a tumor suppressor because of its negative regulation of cell proliferation and induction of apoptosis. For example, large-scale sequencing identified somatic mutations in the p38 MAPK pathway in human tumors [25]. HCC cells were reported to have lower levels of p38 and MKK6 activities than normal liver cells, implying that increased p38 activity induces apoptosis in hepatoma cell lines [26]. Importantly, increased liver tumorigenesis was observed in p38-deficient mice [27]; these mice are also highly sensitive to K-Ras-induced lung tumorigenesis [28]. Therefore, therapeutic approaches that can reactivate p38 in cancer cells have potential to inhibit tumor growth.

In conclusion, our study provided novel insights into the mechanisms underlying the regulation of apoptosis by ATAD2 in HCC cells. High levels of ATAD2 in HCC cells suppress p53- and p38-mediated apoptosis, promoting tumor growth. The direct interactions of ATAD2 with MKK3/6 prevent p38 activation, and contribute to tumor progression. Thus, therapeutic approaches aimed at neutralizing ATAD2 activity are likely to be effective anti-cancer strategies. Indeed, ATAD2 is a highly druggable target, and recent efforts using a fragment-based discovery approach have identified potential inhibitors of the ATAD2 bromodomain [29]. Additional approaches may include specific inhibition of ATAD2-MKK3/6 interaction, which have the potential to reactivate p38 tumor suppressor function, and may benefit the large percentage of HCC patients with mutant p53. Taken together, our data suggest that ATAD2 is a clinically and biologically relevant therapeutic target in HCC which is amenable to the development of effective anti-cancer approaches.

## MATERIALS AND METHODS

### Patient samples

Human HCC and their adjacent non-tumor liver tissue samples were obtained during routine surgical procedures performed at Queen Mary Hospital, the University of Hong Kong, from 1993 to 2003 and at Stanford Hospital, Stanford University, from 2009 to 2012. The studies were approved by the Institutional Review Boards at both institutions for the use of human subjects in medical research. Cohort 1 included 75 pairs of HCC tumor tissue and their matched non-tumor tissue that were surgically removed and snap-frozen in liquid nitrogen for RNA extraction. Microarray analyses were performed, and results described in Chen *et al* [16]. Gene expression data of ATAD2 were extracted from the original data described in Chen *et al.* An independent Cohort 2 included 82 cases of archived HCC samples retrieved from Surgical Pathology at Stanford Hospital.

### Real-time semi-quantitative PCR

Total RNA was extracted from 51 HCC tissues and 27 adjacent non-tumor tissues using RNeasy Mini Kit (Qiagen, Valencia, CA), and 1 μg total RNA from each sample was reverse transcribed using the High-Capacity cDNA Reverse Transcription Kit (Invitrogen, Carlsbad, CA). Real-time q-PCR analysis was done using Taqman probes for ATAD2 and 18S in a 7500 Fast Real-Time q-PCR system (Applied Biosystems Inc., Foster City, CA). All reactions were done in duplicates in three independent assays under the following cycling conditions: 50°C for 2 min, 95°C for 10 min, 40 cycles of 95°C for 15 s and 60°C for 1 min. Relative transcriptional fold changes were calculated as 2^−▲▲CT^.

### Immunohistochemistry

Immunohistochemistry was performed as previously described with minor modifications [30]. Tissue sections were deparaffinized by submerging in xylene and rehydrated in decreasing concentrations of ethanol (100% followed by 90% and 75%, twice at each alcohol concentration). For antigen retrieval, the slides were immersed in 10 mmol/L citrate buffer (pH 6.0) and microwave for 15 mins. After washing four times with PBS for 5 mins each, the sections were treated with 3% H_2_O_2_ for 30 minutes and then 10 % normal goat serum for 1 hour. Anti-ATAD2 (Abcam, Cambridge, UK) or p-p38 (Cell Signaling Technology Inc., Danvers, MA) at 1:100 dilution were incubated with slides at 4°C overnight in a humidified chamber. Thereafter, secondary antibodies were added and incubated at room temperature for 1 hour. After washing, sections were developed using Dako EnVision system and were counterstained with hematoxylin (Dako, Glostrup, Denmark). Images were taken with Nikon Epifluorescent upright microscope E600 (Nikon, Tokyo, Japan). Over-expression of ATAD2 in tumor samples was defined as positive cells in > 50% of the entire area.

### Cell lines and cell culture

Human HCC cell lines, HepG2, Hep3B, Huh7, PLC/PRF/5, HepG2-*Luc*(+), and Hep3B-*Luc*(+) were cultured in Dulbecco's Modified Eagle's Medium-high glucose (DMEM) containing 10% fetal bovine serum (FBS), and 1% penicillin-streptomycin in a humidified atmosphere with 5% CO_2_ at 37°C. All media and supplements were obtained from Invitrogen (Invitrogen, Carlsbad, CA).

### siRNA transfection treatment with chemical inhibitors

Target-specific siRNA (Ambion, Austin, TX; 20 nM each) was transfected into HCC cells using RNAimax transfection reagent (Invitrogen, Carlsbad, CA) according to manufacturer's instructions. The protein expression levels before and after siRNA transfection were detected by Western blotting for each target. The siRNA sequences are: ATAD2 siRNA-1: 5- GCG UCG AAG UUG UAG GAU Utt-3; ATAD2 siRNA-2: 5- GCA AGA CCA AGA UAC CGA Utt-3; MKK-3 siRNA: 5- CCC GGA CCU UCA CCA Utt-3; MKK-6 siRNA: 5- GGA UAC AUC ACU AGA UAA Utt-3.

For treatment with chemical inhibitors, cells were first transfected with ATAD2 siRNA-1, and then 5 μM PFT-α, 1 μM PFT-μ, or 5 μM p168316 (Sigma-Aldrich, St. Louis, MO) was added into cell culture medium and co-incubated for 72 hours.

### Protein extraction and western blotting

Tissues or cells were lysed with T-PER Tissue Protein Extraction Reagent (Thermo Fisher Scientific Inc., Rockford, IL) for 15 min on ice, and then sonicated with power 1 for 2 seconds. Supernatants were collected for measurement of protein concentration. Protein lysates (10 μg) were suspended in loading buffer and separated on 10% SDS-polyacrylamide gels and then transferred onto nitrocellulose membranes for incubation with primary antibodies overnight at 4°C. This was followed by incubation with HRP-conjugated secondary antibodies. Immuno-complexes were detected by Supersignal West Pico Chemiluminescent Substrate (Thermo Fisher Scientific Inc., Rockford, IL) according to the manufacturer's protocol. Primary antibodies were: ATAD2, c-PARP, p-p53, Puma, Bax, Bad, Bak, Bim, and Bcl-xL (Cell Signaling Technology Inc., Danvers, MA); p38, MKK3/6, p-MKK3/6, GAPDH (Santa Cruz Biotechnology, Santa Cruz, CA) and Actin (Sigma-Aldrich, St. Louis, MO). Secondary antibodies were: goat anti-rabbit and goat anti-mouse (Santa Cruz Biotechnology, Santa Cruz, CA).

### Cell viability assay

Cells were seeded into 96-well plates at a density of 4 × 10^3^ cells per well. Cell viability was measured using CellTiter 96 Aqueous One Solution Cell Proliferation Assay (Promega, Madison, WI) according to the manufacturer's instructions. Three independent experiments were done, each in triplicates.

### Generation of lentiviral-based shRNA vector and stable cell lines

ATAD2 siRNA-1 or control siRNA were cloned into a doxycycline-inducible *tet-*on lentiviral vector (Ambion, Austin, TX). Constructed lentiviral vectors were generated in HEK293T cells by using Trans-Lenti Packaging Kits (Thermo Fisher Scientific Inc., Rockford, IL). The HepG2, Hep3B, Huh7, PLC/PRF/5, HepG2-*Luc*(+) and Hep3B-*Luc*(+) cells were treated with 2 MOI viral vectors and selected with 3 μg/ml puromycin (Sigma-Aldrich, St. Louis, MO). Cells stably expressing ATAD2-shRNA or control-shRNA were used for colony formation assay, wound healing assay, and xenograft establishment in nude mice.

### Colony formation assay

Cells stably expressing ATAD2 shRNA were seeded at 2.5 × 10^3^ cells per well in 6-well plates. The culture media were changed every 3 days for 2 weeks, and colonies that were formed were stained with crystal violet (Sigma-Aldrich, St. Louis, MO). Images were taken using Epson perfection V500 photo scanner. Triplicate independent experiments were performed, and the number of colonies in three independent wells were counted and presented as mean± SD.

### Wound-healing assay

Hep3B and Huh7 cells stably expressing ATAD2 shRNA were seeded in 6-well plates at 90% confluency. The wound was created using a micropipette tip. The migration of cells towards the wound was monitored every 24 hours and images were taken using Eclipse E600 image analysis system (Nikon, Tokyo, Japan).

### Matrigel invasion assay

The BioCoat Matrigel Invasion Chambers (BD Biosciences, San Jose, CA) were used for invasion assay according to manufacturer's instructions. 2.5 × 10^4^ Cells transfected with ATAD2 siRNA-1 or control siRNA in FBS-free DMEM were added into the top chamber, and DMEM with 10% FBS was added into the bottom chamber. After 24 hours, cells that invaded to the bottom chamber were fixed with methanol and stained with hematoxylin (Sigma-Aldrich, St. Louis, MO). The images were taken using the Eclipse E600 image analysis system (Nikon, Tokyo, Japan). Cell numbers in five continuous fields were counted. The number of cells in each control siRNA group was set as 1.0, and the number of cells in the respective ATAD2 siRNA-treated group was calculated as a ratio compared to control siRNA group.

### Immunofluorescence staining

HCC cells were seeded in chamber slides and fixed with 4% paraformaldehyde in PBS, permeabilized with 0.1% Triton X-100 for 15 min, and immunostained for ATAD2 and MKK3/6 as described [30]. Phalloidin staining was done according to the manufacturer's instructions (Invitrogen, Carlsbad, CA). Images were taken using an Eclipse E600 image analysis system (Nikon, Tokyo, Japan).

### TUNEL assay

Cells were grown on chamber slides and fixed with 4% paraformaldehyde in phosphate-buffered saline (PBS; pH 7.4). The terminal deoxynucleotidyl transferase-mediated dUTP nick end labeling (TUNEL) assay was carried out following the manufacturer's instructions (Roche, Indianapolis, IN). Hematoxylin was used as a counter stain.

### Co-immunoprecipitation assay

Cell lysates (500 μg total protein in 400 μl lysis buffer) were incubated with anti-MKK3/6 antibody at 4°C for 2 hours and then 20 μl of protein A/G PLUS-Agarose beads (Santa Cruz Biotechnology, Santa Cruz, CA) were added and incubated at 4°C overnight. After incubation, the beads were separated from the lysis buffer and washed three times in cold PBS. Immunocomplexes were resuspended in 2x loading buffer, heat-denatured, and centrifuged; supernatants were collected and resolved by SDS-PAGE on a 10% polyacrylamide gel, and immunoblotting was performed with antibodies as indicated.

### Generation and monitoring of subcutaneous xenografts derived from HCC cells with ATAD2 shRNA

HepG2-*Luc*(+) and Hep3B-*Luc*(+) cells stably expressing ATAD2 shRNA or control shRNA (1 × 10^6^ cells) were resuspended in 100 μl matrigel (BD Biosciences, San Jose, CA) and injected into the right or left shoulder respectively, of 4 weeks old nude mice (Charles River Laboratories International Inc., Wilmington, MA) to induce subcutaneous tumor formation (*n* = 5 for each group). Doxycycline (200 μg/ml) was then added into the daily water of nude mice, and the tumor diameters measured weekly using a caliper. The tumor volume was calculated using the formula: volume = (width)^2^ × length/2. The mice were sacrificed on week 4 and the tumor tissues were collected for further analysis.

### Statistical analysis

Data were analyzed using Prism for Windows (version 5.0) (GraphPad Software, La Jolla, CA). For clinic-pathological analysis, Pearson's X^2^ was used to calculate the significance between groups. Categorical data were analyzed *via* Fisher's exact test, whereas an independent *t*-test was used for continuous data. Significance was defined as *p* < 0.05.

## SUPPLEMENTARY MATERIAL FIGURES AND TABLES


