# Supplementary material for: Suppression of ATAD2 inhibits hepatocellular carcinoma progression through activation of p53- and p38-mediated apoptotic signaling
Source: Oncotarget. 2015 Oct 19;6(39):41722–35. doi: 10.18632/oncotarget.6152 (PMC4747184; doi:10.18632/oncotarget.6152)
Supplement: Supplementary file 1 [file oncotarget-06-41722-s001.pdf]

Suppression of ATAD2 inhibits hepatocellular carcinoma progression through activation of p53- and p38-mediated apoptotic signaling

Supplementary Material

Supplementary Table 1. Immunohistochemistry scores [a] for ATAD2 expression levels in 82 HCC patients.

Patient No.	Expression intensity
	ATAD2
1	0
2	0
3	0
4	0
5	0
6	1
7	1
8	1
9	0
10	2
11	2
12	1
13	2
14	2
15	2
16	1
17	2
18	2
19	2
20	1
21	1
22	2
23	2
24	2
25	1
26	2
27	2
28	2
29	2
30	1
31	1
32	2
33	1
34	1
35	2
36	2
37	2
38	2

**39**	**2**
**40**	**1**
**41**	**2**
**42**	**2**
**43**	**2**
**44**	**2**
**45**	**2**
**46**	**2**
**47**	**2**
**48**	**1**
**49**	**2**
**50**	**2**
**51**	**2**
**52**	**1**
**53**	**2**
**54**	**2**
**55**	**2**
**56**	**2**
**57**	**2**
**58**	**2**
**59**	**2**
**60**	**1**
**61**	**1**
**62**	**2**
**63**	**2**
**64**	**0**
**65**	**2**
**66**	**1**
**67**	**1**
**68**	**1**
**69**	**1**
**70**	**1**
**71**	**1**
**72**	**1**
**73**	**1**
**74**	**2**
**75**	**2**
**76**	**2**
**77**	**2**
**78**	**2**
**79**	**2**
**80**	**1**
**81**	**2**
**82**	**1**

[a] 0 – negative; 1– low expression, positive cells present in < 50% of the entire area; 2– high expression, positive cells present in > 50% of the entire area.

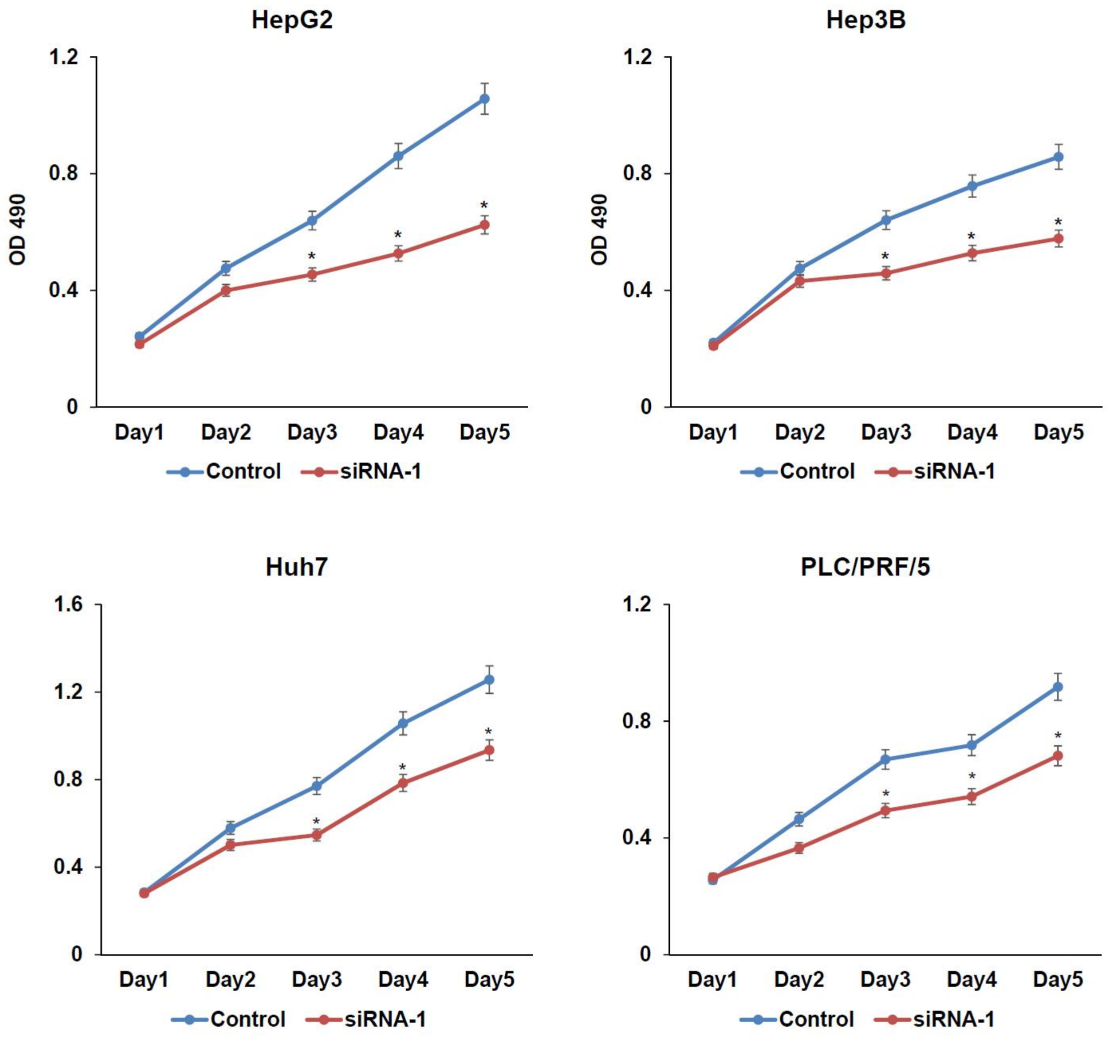

Supplementary Figure 1: ATAD2 suppression in HCC cell lines inhibited cell growth rate. HCC cell lines transiently transfected with ATAD2 siRNA (siRNA-1) showed significantly reduced cell growth rate compared to cells transfected with control siRNA (* $p < 0.05$). Cell viability was assessed daily during a consecutive five-day period, and growth curves plotted.
